# Mental Health of Young Australians during the COVID-19 Pandemic: Exploring the Roles of Employment Precarity, Screen Time, and Contact with Nature

**DOI:** 10.3390/ijerph18115630

**Published:** 2021-05-25

**Authors:** Tassia K. Oswald, Alice R. Rumbold, Sophie G. E. Kedzior, Mark Kohler, Vivienne M. Moore

**Affiliations:** 1 Faculty of Health & Medical Sciences, School of Public Health, The University of Adelaide, Adelaide, SA 5005, Australia; vivienne.moore@adelaide.edu.au; 2 Faculty of Health & Medical Sciences, Robinson Research Institute, The University of Adelaide, Adelaide, SA 5005, Australia; alice.rumbold@sahmri.com (A.R.R.); sophie.kedzior@adelaide.edu.au (S.G.E.K.); 3 South Australian Health & Medical Research Institute, SAHMRI Women and Kids, North Adelaide, SA 5006, Australia; 4 Faculty of Health & Medical Sciences, School of Psychology, The University of Adelaide, Adelaide, SA 5005, Australia; mark.kohler@adelaide.edu.au; 5 The Environment Institute, The University of Adelaide, Adelaide, SA 5005, Australia

**Keywords:** young people, emerging adulthood, mental health, COVID-19 pandemic, screen time, nature, employment, precarity, hope, core beliefs

## Abstract

The coronavirus disease 2019 (COVID-19) pandemic is widely understood to have contributed to mental health problems. In Australia, young people (18–24 years) have been disproportionately affected. To date, research has predominantly focused on the presence or absence of mental illness symptoms, while aspects of mental well-being have been overlooked. We aimed to explore associations between potential risk and protective factors and mental health more comprehensively, using the Complete State Model of Mental Health. An online survey of 1004 young Australians (55% female; M age = 21.23) was undertaken. Assessment of both mental illness and mental well-being enabled participants to be cross-classified into four mental health states. Those with ‘Floundering’ (13%) or ‘Struggling’ (47.5%) mental health reported symptoms of mental illness; a ‘Languishing’ group (25.5%) did not report symptoms of mental illness but mental well-being was compromised relative to those who were ‘Flourishing’ (14%) with high mental well-being. Multinomial logistic regressions were used to examine associations, adjusting for socio-demographic confounders. Protective factors associated with Flourishing mental health included being in secure employment, using screen time to connect with others, and reporting high levels of hope. Both incidental and purposive contact with nature were also associated with Flourishing, while a lack of green/bluespace within walking distance was associated with Languishing, absence of outdoor residential space was associated with Floundering, and lower neighbourhood greenness was associated with all three suboptimal mental health states. Precarious employment, financial stress, living alone, reporting decreased screen time during lockdowns, lower levels of hope, and high disruption of core beliefs were also associated with Struggling and Floundering mental health. Those who were Languishing reported somewhat less hardship and little disruption to core beliefs, but lower levels of hope compared to young people who were Flourishing. This study highlights that young adults require dedicated mental health services to deal with current burden, but should also be supported through a range of preventive strategies which target mental health risk factors, like precarious employment, and enhance protective factors, such as urban green infrastructure.

## 1. Introduction

Impacts of the coronavirus disease 2019 (COVID-19) pandemic have been wide and varied, altering most aspects of daily life. Necessary attempts to curb the spread of COVID-19 through lockdowns, requirements for physical distancing, and restrictions on social gatherings have caused disruptions to employment, education, usual healthcare, and leisure activities [1,2]. Aside from the direct health effects of COVID-19, the mental health consequences of these restrictions and disruptions may be one of the greatest public health impacts of the pandemic [3,4,5]. In Australia, mental health crisis lines have seen significant elevations in calls since the pandemic began and waiting times for mental health services have increased considerably [6,7,8].

The mental health implications of the pandemic appear to be particularly salient for young adults (18–24 years), who are at an important transitional stage of life and face a unique set of challenges [9,10]. They may be in their final year at school, starting or completing tertiary education, and are also more likely to be in precarious employment [11,12,13], so they are at a greater risk of job losses under economic crises like pandemics [14]. Young adults each experience these transitions along different pathways and timelines [15], meaning their social supports and economic circumstances are diverse and fluctuating. For some, this is a time when financial autonomy is established, while for others study or economic insecurity can increase dependence on parental support [16]. Indeed, where pandemic-related research has focused on young adults exclusively, or results have been stratified by age in population-wide surveys, young adults are shown to have experienced higher levels of psychological distress than other age groups during the pandemic, both internationally [17,18,19,20,21] and in Australia [14,22,23,24].

To date, research has highlighted a number of behavioural, lifestyle, socioeconomic, and environmental risk and protective factors for mental illness in the context of the COVID-19 pandemic. These risk and protective factors reflect the daily activities and resources typically available or relevant to young people, but have yet to be fully explored in relation to young adults. In general, unemployment, job loss, and financial stress have consistently shown associations with poorer mental health during the COVID-19 pandemic [2,5,14,22,23,25], but less is known about the influence of job precarity, which, as described earlier, disproportionately affects young people. With more time being spent at home, increased screen time during COVID-19 lockdowns has been reported consistently [19,25,26]. In some studies, this has been associated with poorer mental health [17,27,28,29], with explanations centering around excessive exposure to news cycles, blurred work-life balance as a result of working-from-home, or passive media use leading to lower social support seeking [2,19,26,30]. In other cases, screen time has been linked with better mental health during the COVID-19 pandemic and labelled as a positive coping mechanism which helps individuals stay connected with their communities and social networks [18,26]. Only two of these studies explored the impact of increased screen time among young people specifically [17,18], but both were limited to samples of American college students.

Mobility and other restrictions at different points during the pandemic have also meant that peoples’ immediate physical surroundings are likely to have greater influence on mental health [31]. In particular, having access to public urban greenspaces [32,33,34] and private outdoor spaces [35,36], living in greener neighbourhoods [20,37,38], and having ‘natural’ views from home [36,37,39] have been linked with better mental health across a range of populations during the pandemic. Beyond access and incidental exposure to nature, purposive time spent in nature during the COVID-19 pandemic has also been linked with better mental health [17,20,26,31,34,35,38,39,40,41]. Only two of these studies exploring the influence of nature focused on young people specifically, and were limited to student samples in the USA [17] and Bulgaria [37].

In addition to the aforementioned risk and protective factors, other psychological constructs related to mental health during the COVID-19 pandemic should also be considered. For example, the protective role of hope against anxiety and stress during the pandemic has been demonstrated in research among adults (sample mean age = 37 years) [42]. Contrastingly, in other research among similar aged respondents, the degree to which an individuals’ core beliefs were disrupted by the pandemic explained experiences of depressive and anxiety symptoms to a greater degree than direct (e.g., receiving a COVID-19 diagnosis) and indirect (e.g., loss of child care) pandemic stressors combined [43]. The role of these psychological constructs in young adults’ mental health during the COVID-19 pandemic are not known.

Explorations of risk and protective factors in the context of the COVID-19 pandemic have predominantly focused on the presence or absence of mental illness symptoms, like anxiety and depression. However, mental health does not exist on one continuum with mental illness and mental well-being (absence of mental illness) sitting at opposite ends of the same spectrum. Rather, as the dual-continua model of mental health suggests, mental illness and mental well-being exist on two distinct continua [44,45] and changes in levels of mental well-being are a predictor of future risk of mental illness (e.g., losses of mental well-being predict increases in mental illness, while gains in mental well-being predict declines in mental illness) [46]. Failure to consider this complexity in mental health may mean that adverse consequences for mental well-being, that undermine quality of life without rendering a person mentally ill at the time, are overlooked. As such, there is a need to assess the mental health impacts of the COVID-19 pandemic from a more holistic point-of-view and consider both symptoms of mental illness and symptoms of well-being in conceptualizations of mental health [47]. The Complete State Model of Mental Health provides a more comprehensive perspective of mental health, as it categorizes individuals into four states of mental health: Flourishing (no-to-low mental illness, with high levels of mental well-being), Languishing (no-to-low mental illness, with low levels of mental well-being), Struggling (moderate-to-high mental illness, with high levels of mental well-being), or Floundering (moderate-to-high mental illness, with low levels of mental well-being) [47,48].

Given the independent role of mental well-being, this model is increasingly being applied to study the mental health of young people [47,48,49,50,51,52,53,54,55,56,57,58], but to date has not been applied to understand the impacts of the COVID-19 pandemic on young people’s mental health. The aim of the current study was to explore associations between the four states of mental health and potential risk and protective factors relevant to young Australians and their mental health in the context of the COVID-19 pandemic. We specifically considered factors related to employment and financial security, living arrangements, use of screen time and contact with nature, as well as psychological factors such as level of hope and disruption of core beliefs.

## 2. Materials and Methods

### 2.1. Participants

Participants were recruited through Qualtrics Panels to complete a once-off online survey. To be eligible to participate, individuals had to be living in metropolitan areas of Australia, aged between 18 and 24 years, and proficient in English. The sample was limited to young Australians living in metropolitan areas because impacts of the pandemic, as well as risk and protective factors, are likely to differ considerably for young people living in rural areas. Quota sampling was used in an attempt to capture a sample which covered a spectrum of parameters balanced by gender, state/territory, and socioeconomic status. An area-level indicator of participants’ socioeconomic status (SES), based on residential postcode, was assigned using the Australian Bureau of Statistics (ABS) Socio-Economic Indexes for Areas Index of Relative Advantage and Disadvantage [59]. The ABS scores and ranks geographic areas in Australia on indicators of socioeconomic advantage and disadvantage, based on information gathered in a 5-yearly census. For the purposes of this study, participants were split into quintiles based on these area-level scores (1 = most disadvantaged, 5 = most advantaged).

### 2.2. Measures

#### 2.2.1. Sociodemographic Measures

Participants were asked to provide their age, gender, birthplace, residential postcode, and information about their living arrangement, including type of dwelling (house, townhouse, apartment/unit in single or multi-storey group) and household co-inhabitants (living alone, with a partner, dependent child(ren), parent(s), sibling(s), friend(s) or housemate(s), or others (e.g., extended family members)). Participants indicated whether or not they were doing any formal study or training in 2020 (Year 11 or 12; high school), vocational education and training (VET; workplace-specific often involving apprenticeship), professional development (PD), or university studies). Participants also reported which months of the year they experienced COVID-19 lockdowns.

#### 2.2.2. Mental Well-Being Symptoms

Mental well-being symptoms were measured via the 14-item self-report Mental Health Continuum-Short Form (MHC-SF). The MHC-SF is based on Keyes’ dual continuum theory and measures the three dimensions of well-being: emotional (items 1–3), social (items 4–8), and psychological well-being (items 9–14) [60,61]. Using a 6-point Likert scale (0 = never, 1 = once or twice, 2 = about once a week, 3 = 2 or 3 times a week, 4 = almost every day, 5 = every day), participants indicated how often they had experienced each of the items listed over the last month. Examples of items included feeling “happy”, “satisfied with life” and “that your life has a sense of direction or meaning to it”. Scores on the MHC-SF range from 0–70 and higher scores indicate greater well-being. The scale had a Cronbach’s alpha score of 0.94 in this study.

#### 2.2.3. Mental Illness Symptoms

Mental illness symptoms were measured via the self-report Kessler Psychological Distress Scale (K-10; [62]). This 10-item scale yields a global measure of distress based on questions about depression and anxiety which the respondent has experienced in the past 30 days. Examples of questions include “During the last 30 days, about how often did you feel so nervous that nothing could calm you down?” or “about how often did you feel that everything was an effort?” Response options range from 1 (None of the time) to 5 (All of the time). Scores on the K-10 range from 10–50 and categorise respondents as likely to be well (<20), or having a mild (20 to 24), moderate (25 to 29), or severe (≥30) psychological distress. The K-10 is a widely used measure of psychological distress with high validity, as evidenced in the Australian context [63]. The scale had a Cronbach’s alpha score of 0.92 in this study.

#### 2.2.4. Complete Mental Health States

Each participant was cross-classified into a Complete Mental Health State based on their MHC-SF and K-10 scores (see Table 1 for criteria). As undertaken in previous work by Venning and colleagues [47], pre-determined cut-off scores were used to classify participants as either Flourishing, Languishing, Struggling, or Floundering in life, based on the relative proportion of mental well-being and mental illness symptoms reported. To adapt to the short-form measures used in the current study (in which respondents are positioned within a more compressed range), minor modifications were made to the criteria used by Venning and colleagues [47]. Participants were categorised as (1) *Flourishing in life* if they reported high levels of mental well-being alongside no-to-mild mental illness symptoms; (2) *Languishing in life* if they reported low levels of mental well-being alongside no-to-mild mental illness symptoms; (3) *Struggling in life* if they reported high levels of mental well-being alongside moderate-to-severe mental illness symptoms; or (4) *Floundering in life* if they reported low levels of mental well-being alongside moderate-to-severe mental illness symptoms.

#### 2.2.5. Employment and Financial Variables

Participants were asked to indicate their level of employment precarity in 2020 (permanent, fixed-term contract, regular casual hours, irregular casual hours, receiving JobKeeper payments (welfare support for selected jobs affected by COVID-19 restrictions), or not employed). Individuals who were employed were asked whether they moved to working from home and, if yes, they were asked to indicate on a 5-point Likert scale (1 = Strongly disagree, 5 = Strongly agree) the extent to which they agreed or disagreed with the statement “Working from home has been stressful compared to my usual working arrangements”. Participants were also asked to indicate whether their income and working hours had increased, stayed the same, or decreased as a result of the COVID-19 pandemic.

The InCharge Financial Distress/Financial Well-Being Scale [64] was used to measure participant financial stress. Using a 10-point visual analogue scale (VAS), participants were asked to respond to the question “What do you feel is the level of your financial stress today?” Response options ranged from “No stress at all” to “Overwhelming stress”. Categories were then created to classify participants as having no-to-low financial stress (1–4), moderate financial stress (5–6), or high-to-overwhelming financial stress (7–10).

#### 2.2.6. Screen Time Variables

Participants were asked to indicate whether their overall screen time had increased, stayed about the same, or decreased during COVID-19 lockdowns/restrictions. This was repeated for six specific screen time activities: social media use, video-chatting (e.g., FaceTime, Zoom), streaming services (e.g., Netflix, Stan), video-gaming, phone use, and laptop/computer use. On a 5-point Likert scale (1 = Strongly disagree, 5 = Strongly agree), participants were asked to what degree they agreed with the following statements: “During COVID-19 lockdowns/restrictions: (1) I found technology helpful for staying connected with family and friends, (2) I found myself disengaging from social media or communications over technology (e.g., slower replying to text messages), (3) I felt fatigued by screen time, (4) I felt that technology helped me to cope, (5) I needed to restrict my exposure to news stories in the media”.

#### 2.2.7. Nature Variables

Given there is currently no gold standard for measuring contact with nature [65], access to and incidental contact with nature was gauged through three questions designed specifically for this study. Participants were first asked to indicate whether they had access to a residential outdoor space (no access, balcony, courtyard, or yard). Participants were also asked to indicate whether they lived within walking distance (300 metres according to the World Health Organization [66]) of a greenspace (park, oval, national park) or bluespace (beach, river, lake), and how “green or natural” they perceived their neighbourhood to be on a 10-point VAS (1 = completely urban/built, 10 = completely green/natural).

Purposive contact with nature during COVID-19 lockdowns/restrictions was determined via four questions designed for this study. Participants were first asked to report whether their overall contact with nature had increased, stayed about the same, or decreased during COVID-19 lockdowns/restrictions. This was then repeated for three specific activities: (1) going out in the neighbourhood (walking, jogging, wandering), (2) spending time in a local park, and (3) planning activities in nature (e.g., hiking, picnic, beach walk). Participants were asked to indicate on a 5-point Likert scale (1 = Strongly disagree, 5 = Strongly agree) whether spending time in nature during COVID-19 lockdowns/restrictions (1) gave them a feeling of “getting away”, and whether it (2) felt uncomfortable.

#### 2.2.8. Other Psychological Constructs

Participants’ level of hope was measured via the 12-item Adult Hope Scale (AHS; [67]). Respondents indicate the degree to which each statement describes themselves on an 8-point Likert scale (1 = Definitely false, 8 = Definitely true). Examples of statements include “There are lots of ways around any problem” and “I usually find myself worrying about something.” Scores on the AHS range from 8 to 64 and higher scores indicate a higher level of hope. The scale had a Cronbach’s alpha score of 0.79 in this study.

Individuals each have a broad set of core beliefs which relate to the assumptions they have about themselves, others, the world, and the future. These core beliefs influence how an individual believes others will behave, how events should unfold, and their ability to influence events [68]. Stressful events can sometimes challenge, and cause people to re-examine, their core beliefs. The Core Beliefs Inventory measures the degree to which an individuals’ core beliefs have been disrupted by a stressful event, like the COVID-19 pandemic (CBI; [68]). Participants were asked to reflect upon the COVID-19 pandemic and indicate the extent to which it led them to seriously examine nine core beliefs, on a 6-point Likert scale (0 = Not at all, 5 = To a very great degree). An example of an item is, “Because of the COVID-19 pandemic, I seriously thought about whether things that happen to people are controllable.” Participants’ responses are summed and averaged (final scores ranging from 0 to 5) and higher scores indicate greater disruption of core beliefs. The scale had a Cronbach’s alpha score of 0.87 in this study.

### 2.3. Procedure

The online survey was launched on the 17th of November 2020 and was open until the 9th of January 2021. The survey link was disseminated by Qualtrics to eligible individuals in their double-opt-in research panels. Participants could complete the survey on either a mobile phone or computer device at a time and location of their choice; they were advised that the survey would take 10 to 15 min to complete. All participants provided consent prior to commencing the survey and earned incentive points via Qualtrics Panels for their participation. To guard against duplicate responses, IP filtering was used by Qualtrics. This study was approved by the University of Adelaide School of Psychology Research Ethics Committee (approval number 20/85).

### 2.4. Context

In the lead-up to and during the study period, restrictions were continually changing in Australia in response to the public health recommendations which accompanied COVID-19 outbreak clusters [1]. In the early stages of the pandemic, Australia worked towards reducing the incidence of COVID-19 and “flattening the curve”. In doing so, from March 2020 most states and territories in Australia introduced border restrictions which limited travel across the country, temporary closure of non-essential activities, gatherings and businesses, and people were encouraged to work from home and only go out when essential. Between May and June of 2020, restrictions began to ease across Australia and non-essential services were permitted to operate under new conditions. In late June 2020, stay at home restrictions were reintroduced in the state of Victoria, following a second wave of COVID-19. These restrictions were lifted almost 5 months later in late November 2020, during the study period. Just as this occurred, a cluster outbreak occurred in South Australia which resulted in a 3-day hard lockdown and the closure of several state and territory borders during the study period. A cluster in Northern Sydney (New South Wales) then occurred in December 2020, which resulted in a stay at home order for those areas, new restrictions on social gatherings and non-essential services, and border closures over the week of Christmas. Relative to other countries, Australia has had very few deaths and limited community transmission of COVID-19.

### 2.5. Statistical Analysis

All data were analysed using STATA software version 15.1. Descriptive and bivariate analyses were first conducted to examine relationships between variables. Responses on 5-point Likert scales were recategorized as: “agree” (strongly agree and agree), “neutral” (neither disagree nor agree), and “disagree” (strongly disagree and disagree). Variables were then analysed in a series of multinomial logistic regressions to assess associations between mental health state and factors related to living arrangement, employment, finances, screen time, contact with nature, and hope and core beliefs. Flourishing was used as the outcome reference category and relative risk ratios (RRR) with 95% confidence intervals (95% CI) were calculated. Important relationships between variables were presented in figures.

## 3. Results

### 3.1. Descriptive Statistics

A total of 1004 participants were recruited across seven states and territories in Australia (55% female; M age = 21.23, SD = 1.93). The sample was reasonably well distributed across SES quintiles and 80% of participants were born in Australia. Table 2 presents a summary of sociodemographic variables by mental health state, with bivariate associations shown. Descriptive statistics for all other study variables can be found in the Main Analysis and Table S1.

**Figure 1:**
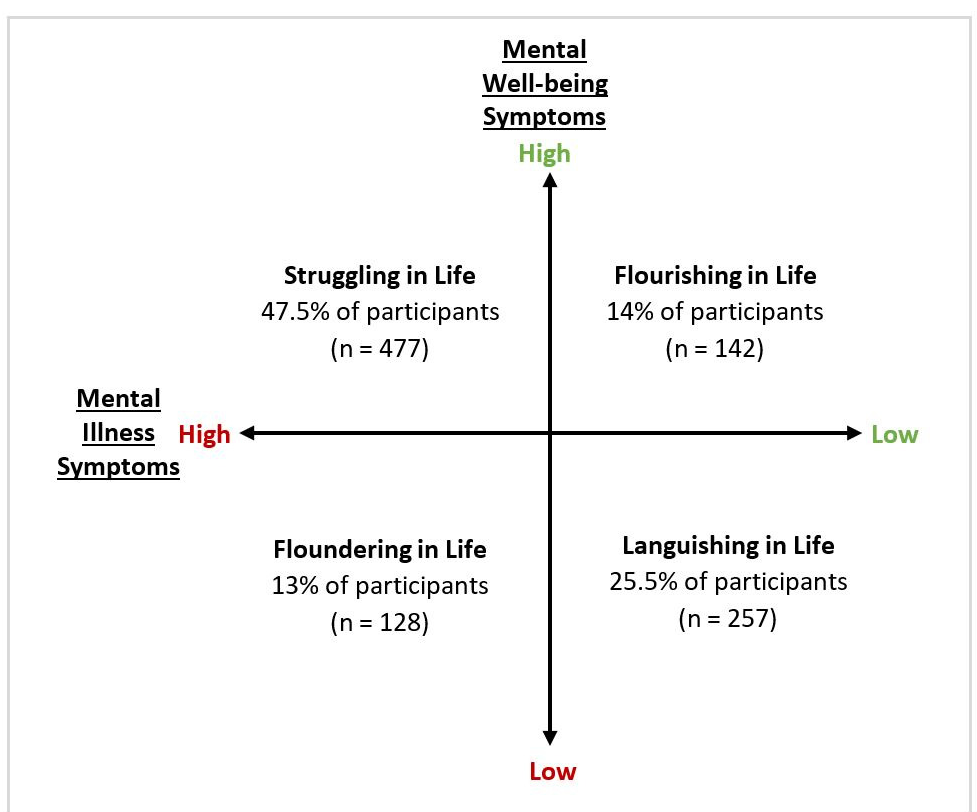
Proportion of sample cross-classified into each of the *Complete State Model of Mental Health* [44,45,47,48] mental health states during the COVID-19 pandemic.

Results on the K-10 indicated that almost one quarter of the sample were likely to be well (n = 228; 23%), while 17% (n = 171), 20% (n = 205), and 40% (n = 401) of participants were classified as experiencing mild, moderate, and severe psychological distress, respectively. Results on the MHC-SF indicated that participants had moderate levels of well-being symptoms on average (M = 36.5, SD = 14.6). After calculating the relative proportion of mental illness and mental well-being symptoms, the largest group in the sample was classified as Struggling in life (n = 477; 47.5%), followed by Languishing (n = 257; 25.5%), Flourishing (n = 142; 14%), and Floundering (n = 128; 13%) (see Figure 1).

### 3.2. Main Analysis

#### 3.2.1. The Role of Living Arrangement, Employment Precarity and Financial Stress

Almost half of participants reported living with parent(s) and/or sibling(s) during 2020 (n = 493; 49%), while only 9% (n = 94) reporting living alone. Associations between young peoples’ living arrangement during the COVID-19 pandemic and mental health state are shown in Table 3. After adjusting for gender, whether young people were studying or employed, and SES, those who lived with their parent(s) and/or sibling(s) or with dependent child(ren) (with or without a partner), were 69% less likely to be Struggling than those who lived alone.

**Figure 2:**
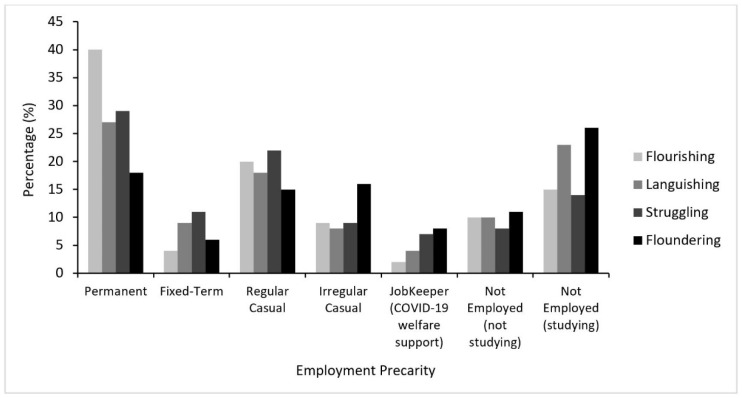
Employment precarity by mental health state during the COVID-19 pandemic.

Across the sample, 29% (n = 288) of participants had permanent employment, while 9% (n = 89) were on fixed-term contracts, 20% (n = 200) worked regular casual hours, and 10% (n = 98) worked irregular casual hours. Five percent (n = 54) of the sample reported that they were on JobKeeper (COVID-19 welfare payments) and 27% (n = 266) reported that they were not employed (n = 176; 66% of those not employed being students). As shown in Figure 2, young people with permanent employment were predominantly Flourishing. In contrast, those who were not employed were predominantly Floundering. Of note, this was also the case for those with irregular casual work and those on JobKeeper.

Associations between employment and financial variables with mental health state are presented in Table 3. Compared to those who had permanent employment, those who were on fixed-term contracts were more than 3 times as likely to be Languishing and Struggling. Young people who worked irregular casual hours were 4 times more likely to be Floundering. Those who were on JobKeeper payments were more than 4 and almost 8 times more likely to be Struggling and Floundering, respectively.

Young people who agreed that working from home was stressful were almost 3 and 5 times more likely to be Struggling and Floundering. Experiencing a decrease or increase in working hours as a result of the COVID-19 pandemic was associated with approximately 2–3 times the risk of Struggling or Floundering, compared to no change in working hours. Compared to reporting no change in income, reporting decreased income as a result of the COVID-19 pandemic was associated with 2.5 times the risk of Struggling and Floundering, while reporting an increase in income was also associated with more than 2 times the risk of Struggling.

**Figure 3:**
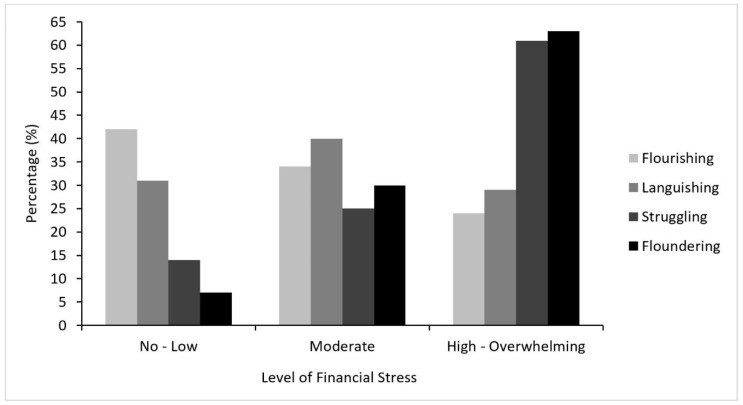
Financial stress by mental health state during the COVID-19 pandemic.

Overall, 48% (n = 478) of the sample reported experiencing high-to-overwhelming levels of financial stress, while 22% (n = 218) reporting experiencing no-to-low financial stress and 31% (n = 308) reported experiencing moderate financial stress. As shown in Figure 3 and Table 3, compared to young people with no-to-low financial stress, those who reported moderate financial stress were over 1.5 times more likely to be Languishing, 2 times more likely to be Struggling, and greater than 5 times more likely to be Floundering. Participants who reported experiencing high-to-overwhelming financial stress were over 7 times more likely to be Struggling and had 15 times the risk of Floundering.

To summarise, living with family, being in permanent employment, and having stable income and working hours were protective factors associated with better mental health during the COVID-19 pandemic (Flourishing). By contrast, living alone, being in precarious employment, experiencing a change in income or working hours, reporting financial stress and stress linked to working from home, were risk factors associated with poor mental health during the COVID-19 pandemic (Languishing, Struggling or Floundering).

#### 3.2.2. The Role of Screen Time

**Figure 4:**
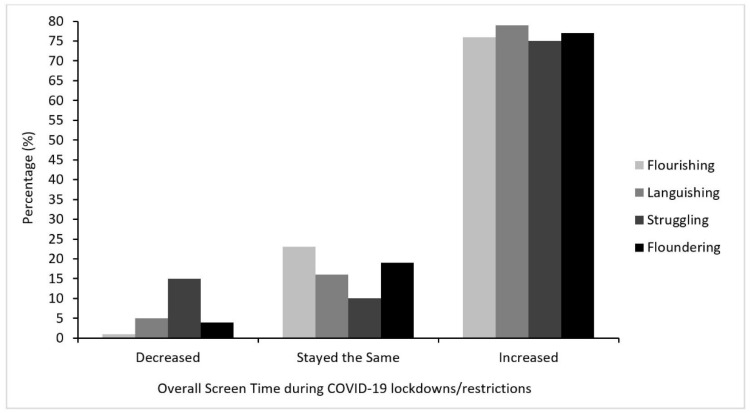
Changes in overall daily screen time during COVID-19 lockdowns/restrictions by mental health state.

The majority of participants reported that their overall screen time had increased during COVID-19 lockdowns/restrictions compared to their typical screen time (77%; n = 769), while 14% (n = 144) reported that their overall screen time stayed about the same. As shown in Figure 4, a small minority of the sample reported decreased screen time (n = 91; 9%). Compared to experiencing typical amounts of screen time during the COVID-19 pandemic, reporting a decreased amount of screen time was associated with almost 24 times the risk of Struggling (see Table 4). Reporting an increased amount of screen time compared to usual was also associated with more than 2 times the risk of Struggling. Similar results were reflected in analyses looking at the different types of screen activities; decreases in each type of screen activity were associated with a greater risk of Struggling, but increases were not (presented in Table S2).

Young people who agreed that screen time helped them connect with family and friends during COVID-19 lockdowns/restrictions were 81% and 76% less likely to be Struggling and Floundering, respectively. Young people who found themselves disengaging from technology-mediated communications were almost 2 times as likely to be Struggling and Floundering. Not feeling fatigued by screen time was associated with Flourishing (50–53% less risk of Languishing and Struggling). Young people who felt as though technology did not help them cope were more than 2.5 times as likely to be Floundering. Feeling the need to restrict exposure to news during COVID-19 lockdowns/restrictions was not independently associated with mental health state.

To summarise, young people with the best mental health (Flourishing) reported that technology helped them cope and connect with family and friends during the pandemic. By contrast, young people with poor mental health reported decreasing their overall screen time during lockdowns (Struggling), alongside experiences of screen time fatigue (Languishing and Struggling) and disengagement from technology-mediated communications (Struggling and Floundering).

#### 3.2.3. The Role of Nature

The majority of the sample had access to nature during the COVID-19 pandemic, with 95% (n = 951) reporting access to a residential outdoor space and 77% (n = 777) having a greenspace or bluespace within walking distance of their home. While 9% (n = 88) of the sample perceived their neighbourhood to be highly natural/green, and 5% (n = 54) perceived their neighbourhood as highly built/urban, the majority of participants reported living in neighbourhoods between the two extremes (e.g., moderately natural, even mix, or moderately built).

Associations between access to nature, incidental contact with nature, and mental health state during the COVID-19 pandemic are shown in Table 5. Compared to having access to a residential outdoor space, young people with no access were 5 times more likely to be Floundering. Living in a neighbourhood which was perceived to be highly built was associated with over 4 times the risk of Floundering, while living in a neighbourhood that was perceived to be highly green/natural was associated with 65% and 75% less risk of Languishing and Floundering, respectively. Compared to having a greenspace and/or bluespace within walking distance of the home, not having this was associated with 1.77 times the risk of Languishing.

**Figure 5:**
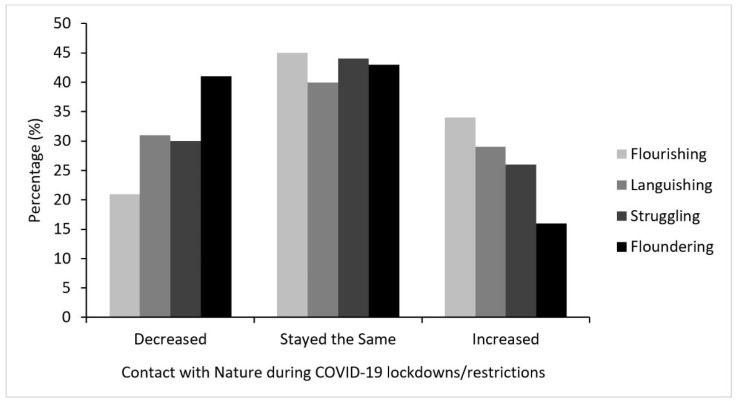
Purposive contact with nature during COVID-19 lockdowns/restrictions by mental health state.

The majority of young people reported that their contact with nature stayed about the same during COVID-19 lockdowns/restrictions (43%; n = 407), while 26% (n = 249) reported that their contact with nature increased and 31% (n = 288) reported a decrease in their contact with nature. As shown in Figure 5, Floundering was the predominant mental health state among those who reported decreased contact with nature, while Flourishing was predominant among those who reported an increase.

Compared to reporting no change in contact with nature during COVID-19 lockdowns/restrictions, young people who reported a decreased amount of contact were almost 2 times more likely to be Floundering, while young people who reported an increased amount of contact with nature were 51% less likely to be Floundering (see Table 6). These results were largely reflected in analyses looking at different types of nature activities (presented in Table S3).

Compared to those who agreed that spending time in nature during COVID-19 lockdowns/restrictions felt like “getting away”, those who disagreed were more than 3 times as likely to be Languishing, more than 4 times as likely to be Struggling, and almost 6 times as likely to be Floundering. Those who endorsed the statement that spending time in nature during COVID-19 “felt uncomfortable” were over 5 times more likely to be Struggling, compared to those who disagreed.

To summarise, having access to a residential outdoor space, living in a neighbourhood which was perceived to be highly green/natural, reporting increased contact with nature during COVID-19 lockdowns, and experiencing feelings of “getting away” in nature, were protective factors associated with the best mental health during the COVID-19 pandemic (Flourishing). Contrastingly, having no access to a residential outdoor space, living in a neighbourhood which was perceived to be highly built/urban, and reporting decreased contact with nature, were risk factors associated with the worst mental health (Floundering). Those who did not have a greenspace and/or bluespace within walking distance of their home were more likely to be Languishing (no mental illness, but low mental well-being), while those who were Struggling reported feeling uncomfortable in nature.

#### 3.2.4. The Role of Other Psychological Constructs

**Figure 6:**
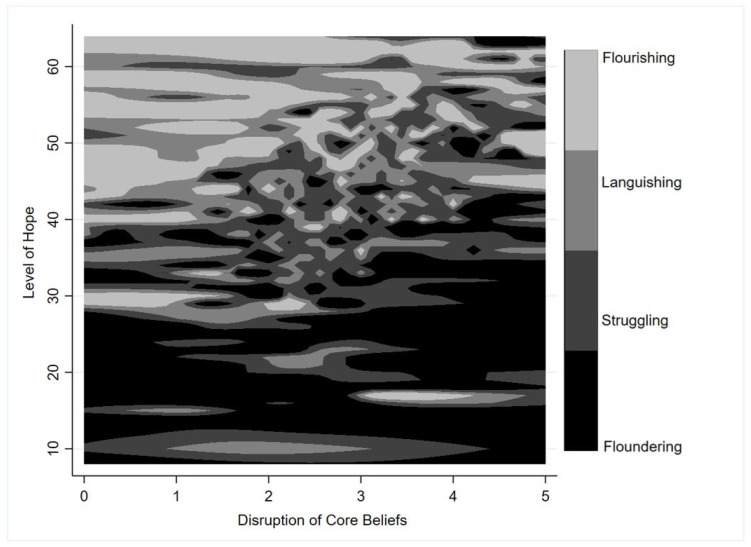
Level of hope and disruption of core beliefs by mental health state during the COVID-19 pandemic. Top left-hand corner corresponds to high levels of hope and low disruption of core beliefs. Bottom right-hand corner corresponds to low levels of hope and high disruption of core beliefs. Greyscale represents the four mental health states.

Overall the sample had a moderate level of hope (M = 42.50, SD = 9.40, range = 8–64) and experienced moderate disruption of core beliefs as a result of the pandemic (M = 2.83, SD = 0.95, range = 0–5). As shown in Figure 6, those who were Flourishing tended to have higher levels of hope (M = 49.83, SD = 7.42, range = 17–64) and lower disruption of their core beliefs (M = 2.80, SD = 1.08), while those who were Floundering tended to have lower levels of hope (M = 33.82, SD = 10.66, range = 8–57) and greater disruption of their core beliefs (M = 2.92, SD = 1.11).

Associations between hope, disruption of core beliefs, and mental health state during the COVID-19 pandemic are shown in Table 7. Each unit increase in hope score was associated with 10%, 15%, and 24% less risk of Languishing, Struggling, and Floundering, respectively. Each unit increase in CBI score (indicating greater disruption of core beliefs as a result of the pandemic) was associated with almost double the risk of Struggling and almost three times the risk of Floundering.

## 4. Discussion

Using the Complete State Model of Mental Health, we explored associations between a number of risk and protective factors and mental health among over 1000 young Australians in the context of the COVID-19 pandemic. A small proportion of this sample were considered Flourishing (14%), with high levels of mental well-being and low-to-mild levels of mental illness symptoms. The largest group in the sample (47.5%) were classified as Struggling, meaning they tended to have moderate-to-high levels of mental well-being, while also experiencing moderate-to-severe psychological distress. This is consistent with most international [17,18,19,20] and Australian [14,22,23,24] literature, which indicates that young adults have experienced high levels of psychological distress during the pandemic. A smaller, yet sizeable proportion of the sample (25.5%), were found to be Languishing, meaning they reported no-to-mild levels of psychological distress, but they also reported low levels of mental well-being. Given levels of mental well-being have been found to predict future levels of mental illness [46], this may have implications for the mental health of this sub-group beyond the pandemic. Overall, the mental health profiles obtained in the current study suggest that young adults should be a target group for both provision of mental health services and preventive strategies in the immediate post-pandemic context.

In promoting the mental well-being of young adults, and reducing future burden of mental illness, this study has identified a range of relevant risk and protective factors. Foremost, advocating for job security is important for promoting mental well-being and preventing mental illness for young adults [69]. While other research has highlighted the mental health impacts of unemployment and job loss during the COVID-19 pandemic [14,25], our study extends this literature and demonstrates the mental health risks of precarious employment. Young people in our sample who had secure employment (e.g., permanent positions) had the best mental health (i.e., Flourishing). By comparison, those in less secure employment (e.g., casual workers) with fewer benefits like sick leave or paid time off for quarantine purposes, had poorer mental health (i.e., Languishing, Struggling, and Floundering). This is concerning for young people globally because they are more likely to be in precarious employment [12,13].

While financial stress due to the pandemic, rather than job loss itself, was reported to be a key correlate of psychological distress in another Australian study [22], our study demonstrated that simply guaranteeing young adults’ income (e.g., through government subsidies), or increasing their working hours, may not counteract distress around employment disruption. Young Australians in our sample who reported an increase in their income as a result of the pandemic were still 2 times more likely to be classified as Struggling than respondents who experienced no change in income. This is similar to those who reported reduced income due to the COVID-19 pandemic, and suggests that instability and changes out of one’s control may be a greater source of distress than currently recognised. Related to this, instability during the pandemic appeared to be associated with worst mental health for young people who were living independently and did not have the social supports or “buffers” available to those living with their parent(s) or partner.

Contemporary technologies have useful functions which can enable important aspects of our social, educational, and occupational lives to continue in the context of the pandemic [26]. While excessive screen time has repeatedly been linked with poorer psychological outcomes in a pre-COVID world [65], a number of recent studies have found screen time to be a useful resource for adaptive coping during lockdowns, through positive escapism or community engagement, for example [18,26]. Young Australians in our sample tended to increase their screen time overall during lockdowns/restrictions, but those who decreased their screen time were significantly more likely to be Struggling with their mental health. When asked about their experiences of using screen time during COVID-19 lockdowns/restrictions, those who had the best mental health in our sample (i.e., Flourishing) appeared to view screen time as a useful resource which helped them cope during the pandemic and connect with family and friends (even when accounting for screen time fatigue). Contrastingly, young people who disagreed that screen time helped them cope during the pandemic were over 2.5 times more likely to have the worst mental health (i.e., Floundering) and appeared to experience screen time fatigue and difficulty engaging with technology-mediated communications during lockdowns/restrictions. Exposure to news stories in the media did not seem to independently affect mental health. In the case of the COVID-19 pandemic, it may be that higher levels of screen time reflect greater engagement and connection which supports mental health, while decreases in screen time indicate a group who may have become withdrawn. If this pattern of low engagement and withdrawal is generalizable, this would have important implications for public health messaging and community-based mental health services, which typically assume that people who are struggling can be reached via social media and media mental health campaigns.

Consistent with a growing body of other research, our results suggest that investment in green infrastructure is important for supporting young people’s mental health “in place” during lockdowns [33,70]. A UK-based study reported that not having access to a private outdoor space during the pandemic was associated with greater psychological distress [20], while other studies have highlighted the mental health benefits offered by domestic gardens for both young and older individuals during lockdowns [35,37,71]. Similarly, in our study, not having access to a residential outdoor space during the pandemic was associated with a 5-fold risk of worst mental health (i.e., Floundering) among young Australians. In contrast to residential outdoor space, not having a public green or bluespace within walking distance of the home was associated with a greater risk of Languishing only, suggesting that this type of green amenity may be particularly pertinent to promoting mental well-being. Across a number of studies, general neighbourhood greenery has also been linked with reduced psychological distress [37,39] and greater positive emotions [36,38,39] during the COVID-19 pandemic. Consistent with this, the degree of neighbourhood naturalness was associated with mental health state in our sample, particularly at either extreme, with young Australians living in highly green/natural environments more likely to have the best mental health (i.e., Flourishing) and those living in highly built neighbourhoods more likely have the worst mental health (i.e., Floundering).

The mental health benefits of access to nature during the pandemic have been demonstrated, but this does not always reflect purposive engagement. While increased visitation to urban greenspaces during the pandemic has been reported across a number of studies internationally [34,38,72,73], some studies have reported decreases in the time people spent in urban greenspaces during the COVID-19 pandemic [32,40] due to mobility restrictions and fear of infection. Individuals who had greater nature engagement during the pandemic typically had better mental health [17,20,35,38,41] and often reported that nature was important for supporting their mental health [31,40,73], helping them cope with lockdowns [36,38], and gave them feelings of “being away” [37]. Similarly, in our study, young Australians who increased their time in nature and agreed that spending time in nature during COVID-19 lockdowns/restrictions felt like “getting away”, had the best mental health (i.e., Flourishing). By contrast, young Australians in our study who “felt uncomfortable” in nature during COVID-19 lockdowns/restrictions were more likely to be Struggling, possibly reflecting similar fears about infection in other studies.

Given that urban greenspaces are not equitably distributed across Australia [74], with low-income neighbourhoods having the least access, the results in our study had the potential to be influenced by level of neighbourhood disadvantage. However, even after adjustment for area-level SES, these nature-mental health associations still persisted. Together, these findings highlight the potential mental health implications of high-density living, and emphasise social justice implications of inequitable access to urban greenspaces, especially under extenuating circumstances like pandemics.

The pandemic has shaken many young peoples’ fundamental assumptions about the world, including their beliefs about their personal abilities, their relationships with other people, and their futures more broadly, which can cause considerable distress [43,68]. In times of hardship, hope is a psychosocial resource which can help provide individuals with a means of coping with circumstances out of their control [75]. In our study and others [42,76], hope has shown to be a powerful protective factor for mental health during the pandemic. This is an important public health finding and suggests that population-level mental health interventions should move beyond encouragement of self-care towards actively fostering hope in young people through evidence-based approaches [77].

### Limitations

The results of this study must be considered while appreciating some limitations. First, it is not possible to generate a random sample of young adults for an online survey directly from electronic contact details, due to the lack of a sampling frame. While the electoral roll is a reliable sampling frame, this approach requires posting information to participants which is not the best avenue to engage young people [78]. As such, a convenience sample was used. This approach is acceptable since our aim was to not to make prevalence estimates [79], but rather to explore inter-relationships between key variables. Quota sampling meant that the final sample had strengths in terms of size and diversity. It seems unlikely that the associations reported would be different among young people who did not participate. Research about social and mental health surveys indicates that individuals with severe mental illness are less likely to participate in online surveys than those without such conditions [79], nevertheless, some do so, and that was the case in our study.

Due to the cross-sectional nature of the data, the direction of associations remains uncertain, although in many cases it seems reasonable to presume that mental health state is the outcome. Likewise, causation cannot be claimed and there is likely bi-directionality. Furthermore, the changing context of the COVID-19 pandemic (including cluster outbreaks and snap-lockdowns), and the study period taking place over a holiday period (Christmas and the New Year) may have affected participants’ mental health at the time of response. However, the latter may have had less influence, given the majority of data (98% of responses) were obtained before Christmas. Overall, longitudinal studies are required to investigate the potential direction of causation and to determine the long-term psychological effects of the pandemic.

This study may have been strengthened with additional information around the pre-existing mental health status of participants, as well as other lifestyle factors which impact mental health, such as alcohol consumption and smoking status. We also acknowledge that some relative risk ratio estimates should be interpreted with caution, where wide confidence intervals were present as a result of sparse data. Finally, while the results in our study mirror mental health experiences of young adults in similar high-income countries, it is important to note that our findings may not completely generalise to other contexts because COVID-19 infection rates during the study period were significantly lower in Australia than in other high-income countries such as the USA and UK [80]. Given that Australia has been one of the countries least affected by COVID-19 in terms of morbidity and mortality [80], this makes the psychological impacts on young people all the more notable.

## 5. Conclusions

Young adults experience a variety of unique challenges specific to their transitional stage of life. The COVID-19 pandemic appears to have amplified many of these challenges, especially those around independence and security, which may explain why young adults have experienced disproportionate mental health impacts from the pandemic in Australia. Moving forward, young adults not only require focused funding for mental health services to deal with the current burden, but should also be supported through a range of preventive strategies which target mental health risk factors and enhance protective factors. This will involve not only individual-level intervention, but also support for significant structural changes around the way young people work, the environments in which they live, and the way they are able to participate in society more widely.

## Figures and Tables

**Table 1:** Criteria used to categorise participants into complete mental health states.

Mental Health State	K-10 ^a^	MHC-SF ^b^
Flourishing (Complete Mental Health)	Likely to be well (<20) or mild (20–24) psychological distress	Feels 1 of the 3 emotional well-being symptoms “every day” or “almost every day” and feels 6 of the 11 social/psychological symptoms “every day” or “almost every day”
Languishing (Incomplete Mental Health)	Likely to be well (<20) or mild (20–24) psychological distress	Not compatible with Flourishing
Struggling (Incomplete Mental Illness)	Moderate (25–29) or severe (30+) psychological distress	Not compatible with Floundering
Floundering (Complete Mental Illness)	Moderate (25–29) or severe (30+) psychological distress	Feels 1 of the 3 emotional well-being symptoms “never” or “once or twice” and feels 6 of the 11 social/psychological well-being symptoms “never” or “once or twice”

^a^ Kessler Psychological Distress Scale; ^b^ Mental Health Continuum-Short Form.

**Table 2:** Sociodemographic variables by mental health state.

Study Variable	Total n (%)	Flourishing n (%)	Languishing n (%)	Struggling n (%)	Floundering n (%)	*p*-Value
**Age (years)**						0.69
	M = 21.23 (SD 1.93)	M = 21.37 (SD 1.90)	M = 21.37 (SD 1.87)	M = 21.14 (SD 1.20)	M = 21.09 (SD 1.92)	
**Gender**						0.19
Male	450 (45%)	61 (43%)	226 (47%)	115 (45%)	48 (38%)	
Female	548 (55%)	81 (57%)	246 (52%)	141 (55%)	80 (62%)	
Gender Diverse/Non-Binary *	6 (<1%)	0 (0%)	5 (1%)	1 (<1%)	0 (0%)	
**Birthplace**						0.36
In Australia	801 (80%)	113 (80%)	391 (77%)	197 (82%)	100 (78%)	
Outside Australia	203 (20%)	29 (20%)	86 (23%)	60 (18%)	28 (22%)	
**State/Territory of Residence**						0.98
Australian Capital Territory	15 (1%)	2 (1%)	7 (1%)	4 (2%)	2 (2%)	
New South Wales	330 (33%)	47 (33%)	161 (34%)	87 (34%)	35 (27%)	
Queensland	160 (16%)	22 (15%)	70 (15%)	43 (17%)	25 (20%)	
South Australia	79 (8%)	9 (6%)	40 (8%)	19 (7%)	11 (9%)	
Tasmania	39 (4%)	9 (6%)	16 (3%)	9 (3%)	5 (4%)	
Victoria	274 (27%)	35 (25%)	133 (28%)	68 (26%)	38 (30%)	
Western Australia	107 (11%)	18 (13%)	50 (10%)	27 (11%)	12 (9%)	
**Area-Level Socioeconomic Status Quintile**						0.57
1 (most disadvantaged)	191 (19%)	24 (17%)	101 (21%)	39 (15%)	27 (21%)	
2	149 (15%)	19 (13%)	71 (15%)	39 (15%)	20 (16%)	
3	205 (20%)	32 (23%)	97 (20%)	50 (19%)	26 (20%)	
4	224 (22%)	31 (22%)	93 (20%)	68 (26%)	32 (25%)	
5 (most advantaged)	235 (23%)	36 (25%)	115 (24%)	61 (24%)	23 (18%)	
**Studying in 2020**						0.04
Not studying	343 (34%)	54 (38%)	92 (36%)	153 (32%)	44 (35%)	
Year 11 or 12 (high school)	71 (7%)	7 (5%)	14 (5%)	44 (7%)	6 (5%)	
VET or PD	208 (21%)	27 (19%)	38 (15%)	110 (21%)	33 (26%)	
University	381 (38%)	54 (38%)	112 (44%)	170 (38%)	45 (35%)	
**Type of Residential Dwelling**						0.47
Apartment/Unit (Multi-Storey Group)	135 (13%)	15 (11%)	37 (14%)	73 (15%)	19 (15%)	
Unit (Single-Storey Group)	144 (14%)	16 (11%)	37 (14%)	72 (15%)	10 (8%)	
Town house	88 (9%)	12 (9%)	20 (8%)	44 (9%)	12 (10%)	
House	634 (63%)	98 (69%)	163 (63%)	287 (60%)	86 (68%)	
**Months in COVID-19 lockdowns**						0.34
	M = 3.57 (SD 2.74)	M = 3.73 (SD 2.80)	M = 3.68 (SD 2.69)	M = 3.29 (SD 2.63)	M = 4.20 (SD 3.04)	

M = mean; SD = standard deviation; VET = vocational education and training; PD = professional development; * gender diverse/non-binary participants were not included in gender analysis due to small cell size.

**Table 3:** Associations between living arrangement, employment, and financial variables with mental health state during the COVID-19 pandemic.

Variables	Languishing vs. Flourishing	Struggling vs. Flourishing	Floundering vs. Flourishing
**Living arrangement**	RRR^a^ (95% CI)	RRR^a^ (95% CI)	RRR^a^ (95% CI)
Alone	1.00 (Reference)	1.00 (Reference)	1.00 (Reference)
Couple	0.58 (0.20–1.70)	0.51 (0.19–1.35)	0.67 (0.20–2.19)
Parent(s) and/or sibling(s)	0.55 (0.22–1.37)	**0.31 (0.13–0.71)**	0.43 (0.16–1.19)
Dependent child(ren) (with or without partner)	0.39 (0.11–1.30)	**0.31 (0.11–0.92)**	0.36 (0.09–1.47)
Housemate(s)/Friend(s)	0.85 (0.30–2.46)	0.42 (0.16–1.11)	0.66 (0.20–2.14)
Other mix	0.85 (0.29–2.52)	0.51 (0.19–1.38)	0.66 (0.20–2.23)
**Employment precarity**	RRR^b^ (95% CI)	RRR^b^ (95% CI)	RRR^b^ (95% CI)
Permanent	1.00 (Reference)	1.00 (Reference)	1.00 (Reference)
Fixed-Term	**3.32 (1.27–8.71)**	**3.52 (1.42–8.68)**	2.92 (0.88–9.67)
Regular Casual Hours	1.40 (0.77–2.53)	1.50 (0.88–2.53)	1.65 (0.77–3.55)
Irregular Casual Hours	1.39 (0.62–3.12)	1.39 (0.68–2.86)	**4.02 (1.67–9.67)**
JobKeeper (COVID-19 welfare support)	2.84 (0.74–10.88)	**4.27 (1.25–14.60)**	**7.89 (1.97–31.51)**
Not Employed	**1.99 (1.17–3.38)**	1.17 (0.71–1.93)	**3.22 (1.67–6.23)**
**Working from home was stressful**	RRR^b^ (95% CI)	RRR^b^ (95% CI)	RRR^b^ (95% CI)
Disagree	1.00 (Reference)	1.00 (Reference)	1.00 (Reference)
Neutral	1.07 (0.38–3.00)	1.31 (0.50–3.41)	2.67 (0.51–14.00)
Agree	1.07 (0.44–2.56)	**2.98 (1.35–6.56)**	**4.58 (1.10–19.06)**
**Change in work hours during COVID-19**	RRR^b^ (95% CI)	RRR^b^ (95% CI)	RRR^b^ (95% CI)
Stayed the same	1.00 (Reference)	1.00 (Reference)	1.00 (Reference)
Decreased	0.96 (0.57–1.61)	**2.61 (1.62–4.20)**	**3.21 (1.61–6.41)**
Increased	0.96 (0.45–2.06)	**3.01 (1.54–5.88)**	**3.47 (1.39–8.64)**
**Change in income during COVID-19**	RRR^b^ (95% CI)	RRR^b^ (95% CI)	RRR^b^ (95% CI)
Stayed the same	1.00 (Reference)	1.00 (Reference)	1.00 (Reference)
Decreased	1.28 (0.74–2.21)	**2.53 (1.54–4.15)**	**2.54 (1.32–4.89)**
Increased	1.19 (0.63–2.26)	**2.11 (1.19–3.75)**	1.09 (0.47–2.57)
**Financial Stress**	RRR^b^ (95% CI)	RRR^b^ (95% CI)	RRR^b^ (95% CI)
No-to-Low	1.00 (Reference)	1.00 (Reference)	1.00 (Reference)
Moderate	**1.63 (1.01–2.64)**	**2.10 (1.29–3.40)**	**5.33 (2.35–12.10)**
High-to-Overwhelming	1.66 (0.98–2.82)	**7.27 (4.42–11.97)**	**15.28 (6.81–34.30)**

RRR^a^ = relative risk ratio adjusted for gender, studying (yes/no), employed (yes/no), and socioeconomic status (SES); RRR^b^ = relative risk ratio adjusted for gender, studying (yes/no), and SES; 95% CI = 95% confidence interval; statistically significant associations bolded.

**Table 4:** Associations between screen time variables and mental health state during COVID-19 lockdowns/restrictions.

Variables	Languishing vs. Flourishing	Struggling vs. Flourishing	Floundering vs. Flourishing
**Change in screen time**	RRR^a^ (95% CI)	RRR^a^ (95% CI)	RRR^a^ (95% CI)
Stayed the same	1.00 (Reference)	1.00 (Reference)	1.00 (Reference)
Decreased	4.53 (0.95–21.72)	**23.85 (5.44–104.41)**	3.27 (0.58–18.37)
Increased	1.42 (0.83–2.37)	**2.20 (1.32–3.65)**	1.18 (0.65–2.17)
**Experience variables**	RRR^b^ (95% CI)	RRR^b^ (95% CI)	RRR^b^ (95% CI)
Screen time helped connect with family and friends						
Neutral	1.00 (Reference)	1.00 (Reference)	1.00 (Reference)
Disagree	0.56 (0.18–1.76)	0.55 (0.19–1.56)	0.72 (0.22–2.34)
Agree	0.55 (0.22–1.36)	**0.19 (0.08–0.43)**	**0.24 (0.09–0.62)**
Found myself disengaging from technology communications						
Neutral	1.00 (Reference)	1.00 (Reference)	1.00 (Reference)
Disagree	1.12 (0.66–1.91)	0.74 (0.44–1.25)	**0.43 (0.20–0.90)**
Agree	1.15 (0.67–1.99)	**1.76 (1.07–2.89)**	**1.92 (1.05–3.51)**
Screen time was fatiguing						
Neutral	1.00 (Reference)	1.00 (Reference)	1.00 (Reference)
Disagree	**0.50 (0.26–0.97)**	**0.47 (0.25–0.89)**	0.51 (0.22–1.16)
Agree	1.11 (0.63–1.96)	1.67 (0.97–2.88)	1.29 (0.66–2.51)
Technology helped me cope						
Neutral	1.00 (Reference)	1.00 (Reference)	1.00 (Reference)
Disagree	1.24 (0.57–2.72)	1.44 (0.67–3.09)	**2.56 (1.06–6.14)**
Agree	0.65 (0.39–1.07)	1.08 (0.67–1.76)	0.96 (0.52–1.76)
Needed to restrict exposure to news						
Neutral	1.00 (Reference)	1.00 (Reference)	1.00 (Reference)
Disagree	0.96 (0.52–1.76)	0.69 (0.38–1.24)	0.60 (0.28–1.27)
Agree	1.05 (0.61–1.84)	1.08 (0.64–1.82)	0.78 (0.41–1.48)

RRR^a^ = relative risk ratio adjusted for gender, studying (yes/no) and SES; RRR^b^ = relative risk ratio adjusted for gender, studying (yes/no), SES, and other screen time experience variables in the table; 95% CI = 95% confidence interval; statistically significant associations bolded.

**Table 5:** Associations between access to nature, incidental contact with nature, and mental health state during the COVID-19 pandemic.

Variables	Languishing vs. Flourishing	Struggling vs. Flourishing	Floundering vs. Flourishing
**Access to residential outdoor space**	RRR^a^ (95% CI)	RRR^a^ (95% CI)	RRR^a^ (95% CI)
Yes	1.00 (Reference)	1.00 (Reference)	1.00 (Reference)
No	0.98 (0.24–4.05)	3.22 (0.95–10.86)	**5.02 (1.35–18.63)**
**Perceived neighbourhood naturalness**	RRR^a^ (95% CI)	RRR^a^ (95% CI)	RRR^a^ (95% CI)
Highly built	1.95 (0.61–6.25)	2.00 (0.65–6.12)	**4.05 (1.24–13.27)**
Moderately built	1.34 (0.71–2.54)	1.11 (0.60–2.06)	1.31 (0.62–2.74)
Even mix of built and natural	1.00 (Reference)	1.00 (Reference)	1.00 (Reference)
Moderately green/natural	1.21 (0.74–1.97)	**1.67 (1.07–2.65)**	1.12 (0.63–2.01)
Highly green/natural	**0.35 (0.14–0.85)**	1.56 (0.82–2.98)	**0.25 (0.07–0.91)**
**Greenspace and/or bluespace within walking distance**	RRR^a^ (95% CI)	RRR^a^ (95% CI)	RRR^a^ (95% CI)
Yes	1.00 (Reference)	1.00 (Reference)	1.00 (Reference)
No	**1.77 (1.02–3.06)**	1.62 (0.97–2.73)	1.47 (0.78–2.77)

RRR^a^ = relative risk ratio adjusted for gender, SES and other nature variables in the table; 95% CI = 95% confidence interval; statistically significant associations bolded.

**Table 6:** Associations between purposive nature contact/experiences and mental health state during COVID-19 lockdowns/restrictions.

Variables	Languishing vs. Flourishing	Struggling vs. Flourishing	Floundering vs. Flourishing
**Change in contact with nature during COVID-19**	RRR^a^ (95% CI)	RRR^a^ (95% CI)	RRR^a^ (95% CI)
Stayed the same	1.00 (Reference)	1.00 (Reference)	1.00 (Reference)
Decreased	1.66 (0.97–2.83)	1.46 (0.89–2.39)	**1.98 (1.09–3.58)**
Increased	0.96 (0.59–1.57)	0.79 (0.51–1.24)	**0.49 (0.26–0.95)**
**Experience variables**	RRR^b^ (95% CI)	RRR^b^ (95% CI)	RRR^b^ (95% CI)
Spending time in nature felt like “getting away”					
Disagree	**3.22 (1.18–8.76)**	**4.35 (1.67–11.33)**	**5.92 (2.06–17.03)**
Neutral	1.70 (0.95–3.05)	1.51 (0.87–2.63)	1.88 (0.95–3.70)
Agree	1.00 (Reference)	1.00 (Reference)	1.00 (Reference)
Spending time in nature felt uncomfortable					
Disagree	1.00 (Reference)	1.00 (Reference)	1.00 (Reference)
Neutral	1.39 (0.74–2.59)	**2.61 (1.46–4.69)**	2.10 (1.03–4.25)
Agree	1.35 (0.69–2.63)	**5.51 (3.05–9.94)**	2.32 (1.10–4.89)

RRR^a^ = relative risk ratio adjusted for gender and SES; RRR^b^ = relative risk ratio adjusted for gender, SES, and nature experience variables; 95% CI = 95% confidence interval; statistically significant associations bolded.

**Table 7:** Associations between hope, disruption of core beliefs and mental health state during the COVID-19 pandemic.

Variables	Languishing vs. Flourishing	Struggling vs. Flourishing	Floundering vs. Flourishing
	RRR^a^ (95% CI)	RRR^a^ (95% CI)	RRR^a^ (95% CI)
Increasing Levels of Hope	**0.90 (0.87–0.92)**	**0.85 (0.83–0.88)**	**0.76 (0.73–0.79)**
Increasing Disruption of Core Beliefs	1.06 (0.85–1.32)	**1.97 (1.56–2.48)**	**2.83 (2.04–3.94)**

RRR^a^ = Relative Risk Ratio adjusted for gender, SES, and either hope or disruption of core beliefs respectively; 95% CI = 95% confidence interval; statistically significant associations bolded.

## Data Availability

Participant consent and ethical approval were not obtained to share this data.

## Supplementary Materials

The following are available online at https://www.mdpi.com/article/10.3390/ijerph18115630/s1, Table S1: Descriptive statistics of all study variables, Table S2: Associations between different types of screen activities and mental health state during the COVID-19 pandemic, Table S3: Associations between different types of nature activities and mental health state during the COVID-19 pandemic.
