# Supplementary material for: Mental Health of Young Australians during the COVID-19 Pandemic: Exploring the Roles of Employment Precarity, Screen Time, and Contact with Nature
Source: Int J Environ Res Public Health. 2021 May 25;18(11):5630. doi: 10.3390/ijerph18115630 (PMC8197562; doi:10.3390/ijerph18115630)
Supplement: Supplementary file 1 [file ijerph-18-05630-s001.zip › Table S1.pdf]

Table S1 –

Descriptive statistics of all study variables

Study Variables	Total n = 1,004 (100%)	Flourishing n = 142 (14%)	Languishing n = 257 (26%)	Struggling n = 477 (48%)	Floundering, N = 128 (13%)	p-value
**Employment & Financial Variables**						
Employment Precarity						<.001
Permanent	288 (29%)	57 (40%)	69 (27%)	139 (29%)	23 (18%)	
Fixed-Term	89 (9%)	6 (4%)	24 (9%)	52 (11%)	7 (6%)	
Regular Casual Hours	200 (20%)	28 (20%)	47 (19%)	106 (22%)	19 (15%)	
Irregular Hours	98 (10%)	12 (9%)	21 (8%)	45 (9%)	20 (16%)	
JobKeeper	54 (5%)	3 (2%)	10 (4%)	31 (7%)	10 (8%)	
Not Employed	266 (27%)	35 (25%)	83 (33%)	102 (21%)	46 (37%)	
Working from home was stressful						.001
Disagree	77 (24%)	18 (39%)	25 (36%)	31 (17%)	3 (13%)	
Neutral	60 (19%)	10 (22%)	16 (23%)	29 (16%)	5 (22%)	
Agree	181 (57%)	18 (39%)	29 (41%)	119 (67%)	15 (65%)	
Hours change						<.001
Decreased	373 (50%)	45 (41%)	70 (40%)	211 (56%)	47 (59%)	
Stayed the same	238 (32%)	50 (46%)	82 (47%)	89 (24%)	17 (21%)	
Increased	131 (18%)	14 (13%)	23 (13%)	78 (21%)	16 (20%)	
Income change						<.001
Decreased	317 (43%)	35 (32%)	62 (35%)	177 (47%)	43 (53%)	
Stayed the same	260 (35%)	53 (48%)	76 (43%)	105 (28%)	26 (32%)	
Increased	168 (23%)	22 (20%)	37 (21%)	97 (26%)	12 (15%)	
Financial stress						<.001
No – low	218 (22%)	60 (42%)	80 (31%)	69 (14%)	9 (7%)	
Moderate	308 (31%)	48 (34%)	103 (40%)	118 (25%)	39 (30%)	
High – overwhelming	478 (48%)	34 (24%)	74 (29%)	290 (61%)	80 (63%)	
**Screen Time Variables**						
Overall screen time						<.001
Decreased	91 (9%)	2 (1%)	12 (5%)	72 (15%)	5 (4%)	
Stayed the same	144 (14%)	32 (23%)	42 (16%)	46 (10%)	24 (19%)	
Increased	769 (77%)	108 (76%)	203 (79%)	359 (75%)	99 (77%)	
Social Media Use						<.001
Decreased	105 (11%)	5 (4%)	9 (4%)	78 (17%)	13 (10%)	
Stayed the same	323 (33%)	44 (32%)	92 (36%)	146 (31%)	41 (33%)	
Increased	554 (56%)	88 (64%)	153 (60%)	242 (52%)	71 (57%)	
Videochatting						<.001
Decreased	114 (13%)	5 (4%)	10 (4%)	86 (19%)	13 (13%)	
Stayed the same	302 (34%)	36 (28%)	82 (36%)	150 (34%)	34 (33%)	
Increased	486 (54%)	87 (68%)	138 (60%)	206 (47%)	55 (54%)	
Streaming services						<.001
Decreased	109 (12%)	6 (4%)	13 (5%)	82 (18%)	8 (7%)	
Stayed the same	320 (34%)	42 (31%)	87 (36%)	155 (34%)	36 (31%)	
Increased	512 (54%)	87 (64%)	140 (58%)	214 (47%)	71 (62%)	
Videogaming						<.001
Decreased	148 (19%)	10 (9%)	25 (13%)	102 (26%)	11 (12%)	
Stayed the same	303 (38%)	45 (41%)	78 (40%)	147 (37%)	33 (36%)	

Increased	346 (43%)	56 (50%)	93 (47%)	150 (38%)	47 (52%)	
Phone Use						<.001
Decreased	110 (11%)	1 (<1%)	11 (4%)	92 (20%)	6 (5%)	
Stayed the same	294 (30%)	42 (30%)	85 (33%)	128 (28%)	39 (31%)	
Increased	570 (59%)	96 (69%)	158 (62%)	235 (52%)	81 (64%)	
Laptop / Computer Use						<.001
Decreased	106 (11%)	1 (<1%)	11 (4%)	85 (19%)	9 (7%)	
Stayed the same	303 (32%)	43 (31%)	89 (36%)	133 (30%)	38 (32%)	
Increased	545 (57%)	94 (68%)	148 (60%)	230 (51%)	73 (61%)	
ST helped stay connected to family & friends						<.001
Disagree	119 (12%)	12 (9%)	23 (9%)	66 (14%)	18 (14%)	
Neutral	143 (15%)	7 (5%)	26 (10%)	87 (19%)	23 (18%)	
Agree	722 (73%)	119 (86%)	205 (81%)	312 (67%)	86 (68%)	
I found myself disengaging from technology						<.001
Disagree	237 (24%)	47 (35%)	83 (33%)	91 (20%)	16 (13%)	
Neutral	327 (34%)	47 (35%)	89 (35%)	146 (32%)	45 (36%)	
Agree	412 (42%)	42 (31%)	83 (33%)	223 (48%)	64 (51%)	
ST was fatiguing						<.001
Disagree	177 (18%)	43 (31%)	49 (19%)	66 (14%)	19 (15%)	
Neutral	255 (26%)	33 (24%)	72 (28%)	114 (24%)	36 (28%)	
Agree	558 (56%)	61 (45%)	135 (53%)	289 (62%)	73 (57%)	
Technology helped me cope						0.06
Disagree	142 (14%)	14 (10%)	38 (15%)	64 (14%)	26 (20%)	
Neutral	290 (29%)	37 (27%)	87 (34%)	128 (28%)	38 (30%)	
Agree	553 (56%)	88 (63%)	129 (51%)	272 (59%)	64 (50%)	
Felt the need to restrict news viewing						<.001
Disagree	221 (23%)	47 (34%)	70 (27%)	80 (17%)	24 (19%)	
Neutral	285 (29%)	37 (27%)	71 (28%)	133 (29%)	44 (35%)	
Agree	476 (48%)	54 (39%)	114 (45%)	249 (54%)	59 (46%)	
**Nature Access Variables**						
Perceived neighbourhood naturalness						<.001
Highly built	54 (5%)	5 (4%)	14 (5%)	21 (4%)	14 (11%)	
Moderately built	140 (14%)	19 (13%)	44 (17%)	56 (12%)	21 (16%)	
Even mix of built and natural	331 (33%)	54 (38%)	91 (35%)	139 (29%)	47 (37%)	
Moderately natural	391 (39%)	48 (34%)	99 (39%)	202 (42%)	42 (33%)	
Highly natural	88 (9%)	16 (11%)	9 (4%)	59 (12%)	4 (3%)	
Greenspace and/or bluespace in walking distance						0.11
No	226 (23%)	21 (15%)	64 (25%)	109 (23%)	32 (25%)	
Yes	777 (77%)	120 (85%)	193 (75%)	368 (77%)	96 (75%)	
Access to residential outdoor space						.004
No	51 (5%)	3 (2%)	6 (2%)	30 (6%)	12 (10%)	
Yes	951 (95%)	139 (98%)	251 (98%)	447 (94%)	114 (90%)	

**Nature Experience Variables**						
Overall contact with nature						.01
Decreased	288 (31%)	29 (21%)	75 (31%)	136 (30%)	48 (41%)	
Stayed the same	407 (43%)	62 (45%)	98 (40%)	196 (44%)	51 (43%)	
Increased	249 (26%)	46 (34%)	70 (29%)	114 (26%)	19 (16%)	
Went out in neighbourhood						.001
Decreased	272 (29%)	33 (24%)	57 (24%)	134 (30%)	48 (44%)	
Stayed the same	366 (39%)	50 (37%)	94 (39%)	183 (41%)	39 (35%)	
Increased	298 (32%)	52 (39%)	91 (38%)	132 (29%)	23 (21%)	
Spent time in local park						.03
Decreased	284 (32%)	37 (28%)	60 (27%)	148 (34%)	39 (39%)	
Stayed the same	371 (42%)	49 (37%)	99 (44%)	179 (41%)	44 (44%)	
Increased	235 (26%)	46 (35%)	66 (29%)	106 (24%)	17 (17%)	
Planned activities in nature						.007
Decreased	305 (34%)	36 (28%)	72 (32%)	149 (34%)	48 (46%)	
Stayed the same	375 (42%)	49 (38%)	103 (45%)	182 (42%)	41 (39%)	
Increased	217 (24%)	45 (35%)	52 (23%)	105 (24%)	15 (14%)	
Time in nature felt like "getting away"						.002
Disagree	109 (11%)	5 (4%)	25 (10%)	62 (14%)	17 (15%)	
Neutral	232 (24%)	23 (17%)	62 (25%)	114 (25%)	33 (28%)	
Agree	613 (64%)	108 (79%)	161 (65%)	278 (61%)	66 (57%)	
Time in nature felt uncomfortable						<.001
Disagree	516 (54%)	103 (75%)	161 (66%)	189 (42%)	63 (55%)	
Neutral	207 (22%)	19 (14%)	51 (21%)	107 (24%)	30 (26%)	
Agree	226 (24%)	15 (11%)	33 (13%)	157 (35%)	21 (18%)	
**Other Psychological Constructs**						
Level of Hope (AHS)						<.001
	M = 42.50 (SD 9.40)	M = 49.83 (SD 7.42)	M = 43.64 (SD 7.97)	M = 41.99 (SD 8.24)	M = 33.82 (SD 10.66)	
Disruption to Core Beliefs (CBI)						<.001
	M = 2.83 (SD 0.95)	M = 2.80 (SD 1.08)	M = 2.58 (SD 0.96)	M = 2.94 (SD 0.83)	M = 2.92 (SD 1.11)	

M = mean; SD = standard deviation; VET = vocational and educational training; PD = professional development; AHS = Adult Hope Scale; CBI = Core Beliefs Inventory.
