# Supplementary material for: Mental Health of Young Australians during the COVID-19 Pandemic: Exploring the Roles of Employment Precarity, Screen Time, and Contact with Nature
Source: Int J Environ Res Public Health. 2021 May 25;18(11):5630. doi: 10.3390/ijerph18115630 (PMC8197562; doi:10.3390/ijerph18115630)
Supplement: Supplementary file 1 [file ijerph-18-05630-s001.zip › Table S2.pdf]

Table S2 –

Associations Between Different Types of Screen Activity and Mental Health State during the COVID-19 pandemic

Variables	Languishing vs. Flourishing RRR[a] (95% CI)	Struggling vs. Flourishing RRR[a] (95% CI)	Floundering vs. Flourishing RRR[a] (95% CI)
**Social Media Use**			
Stayed the same	1.00 (Reference)	1.00 (Reference)	1.00 (Reference)
Decreased	0.86 (0.27 – 2.71)	**4.95 (1.88 – 13.02)**	2.89 (0.94 – 8.83)
Increased	0.82 (0.53 – 1.29)	0.84 (0.55 – 1.28)	0.85 (0.50 – 1.45)
**Video-chatting**			
Stayed the same	1.00 (Reference)	1.00 (Reference)	1.00 (Reference)
Decreased	0.88 (0.28 – 2.77)	**4.04 (1.53 – 10.72)**	2.65 (0.85 – 8.25)
Increased	0.67 (0.42 – 1.08)	**0.56 (0.36 – 0.88)**	0.63 (0.35 – 1.14)
**Streaming services**			
Stayed the same	1.00 (Reference)	1.00 (Reference)	1.00 (Reference)
Decreased	1.05 (0.37 – 2.95)	**3.65 (1.49 – 8.99)**	1.58 (0.50 – 5.01)
Increased	0.78 (0.50 – 1.24)	0.67 (0.44 – 1.03)	0.92 (0.53 – 1.59)
**Video-gaming**			
Stayed the same	1.00 (Reference)	1.00 (Reference)	1.00 (Reference)
Decreased	1.45 (0.64 – 3.31)	**3.00 (1.44 – 6.25)**	1.48 (0.56 – 3.91)
Increased	0.92 (0.56 – 1.52)	0.76 (0.48 – 1.20)	1.13 (0.62 – 2.06)
**Phone Use**			
Stayed the same	1.00 (Reference)	1.00 (Reference)	1.00 (Reference)
Decreased	5.46 (0.68 – 43.70)	**30.71 (4.15 – 227.43)**	6.51 (0.75 – 56.54)
Increased	0.81 (0.52 – 1.27)	0.79 (0.52 – 1.21)	0.88 (0.52 – 1.49)
**Laptop / Computer Use**			
Stayed the same	1.00 (Reference)	1.00 (Reference)	1.00 (Reference)
Decreased	5.37 (0.67 – 43.00)	**27.63 (3.73 – 204.67)**	**10.17 (1.23 – 84.12)**
Increased	0.74 (0.47 – 1.16)	0.75 (0.49 – 1.15)	0.87 (0.51 – 1.48)

ST = screen time; RRR[a] = Relative Risk Ratio adjusted for gender, studying (yes/no) and SES; statistically significant associations bolded.
