# Supplementary material for: Mental Health of Young Australians during the COVID-19 Pandemic: Exploring the Roles of Employment Precarity, Screen Time, and Contact with Nature
Source: Int J Environ Res Public Health. 2021 May 25;18(11):5630. doi: 10.3390/ijerph18115630 (PMC8197562; doi:10.3390/ijerph18115630)
Supplement: Supplementary file 1 [file ijerph-18-05630-s001.zip › Table S3.pdf]

Table S3 –

Associations Between Different Types of Nature Activities and Mental Health State during the COVID-19 pandemic

Variables	Languishing vs. Flourishing RRR[a] (95% CI)	Struggling vs. Flourishing RRR[a] (95% CI)	Floundering vs. Flourishing RRR[a] (95% CI)
**Out in neighbourhood**			
Stayed the same	1.00 (Reference)	1.00 (Reference)	1.00 (Reference)
Decreased	0.93 (0.53 – 1.60)	1.11 (0.68 – 1.82)	**1.85 (1.00 – 3.41)**
Increased	0.93 (0.57 – 1.51)	0.70 (0.45 – 1.10)	0.56 (0.30 – 1.08)
**Time in local park**			
Stayed the same	1.00 (Reference)	1.00 (Reference)	1.00 (Reference)
Decreased	0.82 (0.48 – 1.40)	1.08 (0.67 – 1.75)	0.17 (0.64 – 2.14)
Increased	0.73 (0.44 – 1.21)	0.64 (0.40 – 1.02)	**0.41 (0.20 – 0.81)**
**Planned activities in nature**			
Stayed the same	1.00 (Reference)	1.00 (Reference)	1.00 (Reference)
Decreased	0.95 (0.56 – 1.61)	1.11 (0.68 – 1.79)	1.59 (0.88 – 2.90)
Increased	**0.54 (0.32 – 0.91)**	0.64 (0.40 – 1.03)	**0.40 (0.20 – 0.82)**

RRR[a] = Relative Risk Ratio adjusted for gender and SES; statistically significant associations bolded.
